# Tuning the Liquid–Vapour Interface of VLS Epitaxy for Creating Novel Semiconductor Nanostructures

**DOI:** 10.3390/nano13050894

**Published:** 2023-02-27

**Authors:** Galih R. Suwito, Vladimir G. Dubrovskii, Zixiao Zhang, Weizhen Wang, Sofiane Haffouz, Dan Dalacu, Philip J. Poole, Peter Grutter, Nathaniel J. Quitoriano

**Affiliations:** 1 Department of Mining and Materials Engineering, McGill University, Montreal, QC H3A 0C5, Canada; 2 Faculty of Physics, St. Petersburg State University, St. Petersburg 199034, Russia; 3 Department of Physics, McGill University, Montreal, QC H3A 2T8, Canada; 4 National Research Council Canada, Ottawa, ON K1A0R6, Canada

**Keywords:** VLS growth, semiconductor nanostructures, Au-Si-Ge alloy, incubation time, liquid–vapour interface

## Abstract

Controlling the morphology and composition of semiconductor nano- and micro-structures is crucial for fundamental studies and applications. Here, Si-Ge semiconductor nanostructures were fabricated using photolithographically defined micro-crucibles on Si substrates. Interestingly, the nanostructure morphology and composition of these structures are strongly dependent on the size of the liquid–vapour interface (i.e., the opening of the micro-crucible) in the CVD deposition step of Ge. In particular, Ge crystallites nucleate in micro-crucibles with larger opening sizes (3.74–4.73 μm^2^), while no such crystallites are found in micro-crucibles with smaller openings of 1.15 μm^2^. This interface area tuning also results in the formation of unique semiconductor nanostructures: lateral nano-trees (for smaller openings) and nano-rods (for larger openings). Further TEM imaging reveals that these nanostructures have an epitaxial relationship with the underlying Si substrate. This geometrical dependence on the micro-scale vapour–liquid–solid (VLS) nucleation and growth is explained within a dedicated model, where the incubation time for the VLS Ge nucleation is inversely proportional to the opening size. The geometric effect on the VLS nucleation can be used for the fine tuning of the morphology and composition of different lateral nano- and micro-structures by simply changing the area of the liquid–vapour interface.

## 1. Introduction

The metal-semiconductor(s) eutectic system has been of interest in the modern micro- and nano-electronics industry. It has been used for decades to realize semiconductor nanowires via the vapour–liquid–solid (VLS) process. After the discovery of the VLS growth of Si nanowires in the Au-Si eutectic system by Wagner and Ellis (1964) [1], nanowires made from different eutectic systems have been extensively studied especially those based on group III-V [2,3] and other group-IV semiconductors [4,5] for realizing new opto- and nano-electronic devices with unprecedented performances exploiting their low dimensional structures [6,7]. These all were made possible by the advancement of fundamental understanding of the VLS epitaxial growth process. 

VLS epitaxy uses a metal catalyst (e.g., Au) which forms a eutectic liquid solution with precursor and/or substrate atoms (usually semiconductors) at a growth temperature above the system’s eutectic temperature [1]. In chemical vapour deposition (CVD), the subsequent crystal nucleation, then, preferentially occurs at these eutectic liquid droplets resulting in vertical nanowires with a metal liquid droplet radius [1,8]. The theoretical understanding of this phenomenon was pioneered by Givargizov and Chernov (1973) who were the first to relate the VLS growth rate with the metal catalyst size [8,9], motivated by the experimental observation of Wagner and Ellis [1]. Their formula accounts for the Gibbs–Thomson effect which is dominant at small radii and effectively gives a critical radius of the VLS growth. These observations on the geometrical dependence of the VLS growth at the nanoscale motivated subsequent research in controlling the dimension of the metal nano-droplets, including the use of a growth template [2] and controlling the degree of metal nanodroplets’ agglomeration [10].

In VLS epitaxy, precursor atoms contribute to the growth process via an interaction with the liquid–vapour interface of the metal catalyst. Experiments have shown that different sizes of liquid–vapour interfaces result in different VLS growth rates due to different doses of precursor atoms that reach the metal catalyst, even with the same CVD growth conditions [11]. By tuning the size of the liquid–vapour interface, a solidified eutectic microstructure composed of a metal catalyst and semiconductor(s) with unique (semiconductor) nanostructures can be achieved [12], due to different eutectic compositions [13]. The ability to control and isolate these semiconductor nanostructures is attractive as building blocks for novel opto- and nano-electronic device applications. In addition, a basic understanding of the effects of the liquid–vapour interface on the kinetics of the VLS epitaxy and its resulting nanostructures is important for better control of the VLS epitaxy for various applications, including nanowires growth [14] and VLS-assisted heteroepitaxy [11,13,14,15,16].

In this work, we systematically studied the nanostructures of the solidified Au-Si-Ge eutectic alloy grown in a confined geometry fabricated using a “micro-crucible” to shed light on the effects of liquid–vapour-interface size on the VLS process. In this study, a Si substrate was used due to technological interests to integrate more semiconductor nanostructures into the Si platform. The incorporation of Ge into the eutectic (to create a Au-Si-Ge alloy) was intended as a semiconductor “marker” which is more surface sensitive than Si (the substrate material in this study). More practically, we sought to understand the effects of the size of the liquid–vapour interface on the resulting semiconductor nanostructures within the solidified Au-Si-Ge eutectic alloys. We developed a model, relating the incubation time and the liquid–vapour interface size, to explain the observed strong dependence of the liquid–vapour interface size on the VLS nucleation. 

## 2. Materials and Methods

### 2.1. Fabrication of Micro-Crucible Templates

**Figure 1:**
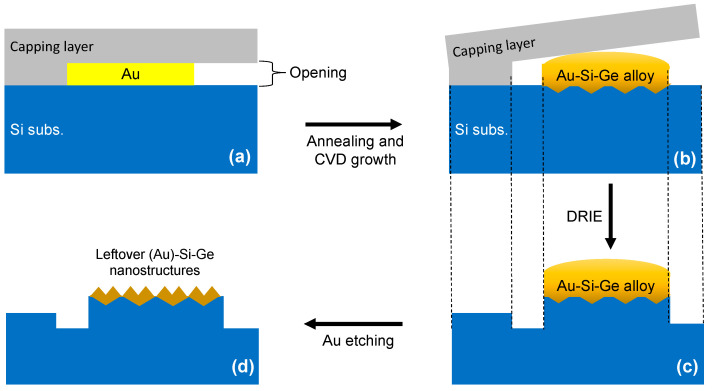
Cross-sectional schematics of the sample preparation process. (**a**) A micro-crucible structure that acted as the growth template where the micro-scale Au seed was placed under a capping layer (made of SiO_2_/Si_3_N_4_) with a well-defined “opening.” (**b**) After annealing at a temperature higher than the Au-Si eutectic temperature of ≅363 °C for 15 min and then exposing the micro-crucible with 4 sccm of GeH_4_ inside a CVD chamber (40 mTorr, 375 °C) for 1 h, a Au-Si-Ge eutectic alloy was formed inside the micro-crucible. At this point, the alloy/Si substrate interface would be rough as the underlying Si surface was dissolved during the eutectic formation. In addition, due to the agglomeration of the Au-Si-Ge eutectic liquid during this step, the capping layer was deformed and the alloy was displaced from its initial position. (**c**) Deep reactive ion etching (DRIE) for 18 min was carried out using SF_6_ to physically remove the capping layer and expose the Au-Si-Ge alloy. The dashed lines indicate the height profile created from the DRIE. (**d**) The sample was then dipped into a Transene Gold Etchant TFA solution for 55 s to remove (the majority of) the Au species from the Au-Si-Ge alloy resulting in the exposure of some semiconductor nanostructures.

In this study, the eutectic alloys of Au-Si-Ge with confined geometry were fabricated by utilizing “micro-crucible” templates made of a photolithographically-patterned SiO_2_/Si_3_N_4_ capping layer on a Si substrate, as schematically shown in Figure 1a, that allows fine control on the geometry of the micro-crucibles via the uses of different photomasks (the microfabrication process of such micro-crucibles can be found in Ref. [11]). In this micro-crucible, the Au catalyst seeds for the VLS growth had micrometer dimensions and were encapsulated selectively by the capping layer with well-defined “openings” (Figure 1a). These openings effectively controlled how many precursor atoms (later in the CVD step) could be reached, and then, they were dissolved into the Au(-Si) catalyst and underwent a VLS growth process after a supersaturation state was reached. Our microfabrication process [11] enables the preparation of such openings with precise geometries and dimensions. Briefly, a photolithography step was completed to pre-pattern micro-scale Au seeds (by e-beam evaporation) on a Si substrate. Then, a ~1.3 μm-thick PECVD-grown capping layer of SiO_2_/Si_3_N_4_ was deposited to encapsulate the Au seeds. Afterwards, an SF_6_-based deep reactive ion etching (DRIE) was used to selectively create openings with the help of another photolithography step.

### 2.2. Preparation of the Au-Si-Ge Eutectic

After the completion of the microfabrication steps [11], the substrate with various micro-crucibles was then inserted into a CVD chamber after sequential cleaning in piranha and in HF solutions to remove organic residues and native oxide, respectively. Then, the sample was annealed at a temperature higher than the Au-Si eutectic temperature of ≅363 °C. This induced the formation of Au-Si eutectic liquid droplets (inside the micro-crucibles) that agglomerated and deformed the capping layer [16]. The agglomeration also made the Au-Si eutectic alloy displace from the initial position of the Au seed [16]. In addition, the interface between the eutectic alloy and the Si substrate became faceted (revealing (111) planes) as the underlying Si was dissolved into the Au during the eutectic formation [17]. After 15 min of annealing, the growth temperature and pressure were set to 375 °C and 40 mTorr, respectively, then GeH_4_ was introduced into the system for 1 h. Figure 1b schematically shows the solidified Au-Si-Ge eutectic alloy after CVD growth. Note that the GeH_4_ precursor gas only reached parts of the Au that were exposed via the pre-defined opening. 

### 2.3. Removal of Au

**Figure 2:**
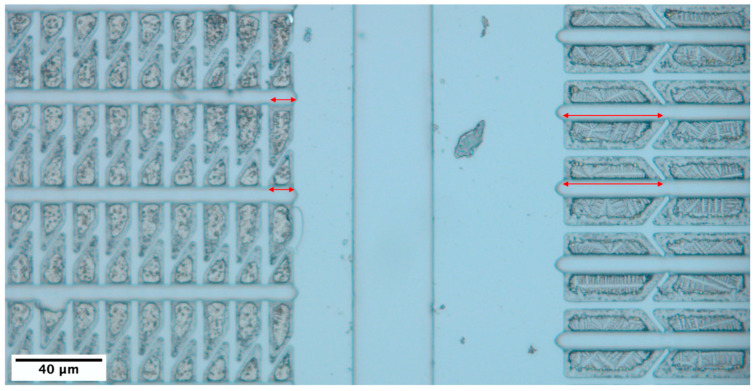
Plan-view optical micrograph of the sample after the removal of the SiO_2_/Si_3_N_4_ capping layer and then the Au species, with small (vertical trapezoids, **left**) and large (horizontal trapezoids, **right**) micro-crucible openings. Different residual micro- and nanostructures can be observed from these two different geometries. The red double arrows indicate where the openings were located.

Here, we studied the semiconductor micro- and nanostructures (from the solidified Au-Si-Ge eutectic alloy) revealed after the removal of (the majority of) the Au. For this reason, after introducing Ge into the Au-Si eutectic alloy via a CVD growth step, we etched back the capping layer with DRIE using SF_6_, a blanket etching without masking. This DRIE was carried out for 18 min (over-etched) to ensure the capping layer was removed. Figure 1c schematically shows the sample after this long DRIE. As shown in the figure, at this point all Au-Si-Ge eutectic alloys were exposed (uncapped). Since the SF_6_ gas also attacks the Si substrate [18], this over-etching step created height profiles on the Si substrate, as schematically indicated by the dashed lines in Figure 1b,c. Afterward, the sample was dipped into the Transene Gold Etchant TFA solution for 55 s to remove (most of) the Au species from the eutectic alloy, as schematically shown in Figure 1d. The final sample under an optical microscope is shown in Figure 2.

### 2.4. Characterization Methods

In addition to an optical microscope (Olympus MX40, Olympus, Tokyo, Japan), a scanning electron microscope (SEM, Hitachi SU3500, Hitachi, Tokyo, Japan) was used to characterize the micro- and nanostructures of the solidified Au-Si-Ge eutectic alloy. During the SEM measurements, an accelerating voltage of 20 kV was used. The chemical compositions of the micro- and nanostructures were characterized using energy-dispersive X-ray spectroscopy (EDS) coupled to the SEM with the same accelerating voltage of 20 kV. For all of the EDS measurements, the working distance was set to 10 mm. Finally, the regions of interest were also examined with an Asylum Research MFP-3D atomic force microscope (AFM, Asylum Research, Santa Barbara, CA, USA) in AC mode. The cross-sectional TEM lamella was prepared using a Hitachi Ethos NX5000 Focus Ion beam scanning electron microscope (FIB-SEM, Hitachi, Tokyo, Japan). Then, the TEM measurement was carried out with Thermo Scientific Talos F200X G2 (S)TEM (Thermo Fisher Scientific, Waltham, MA, USA).

## 3. Results and Discussion

Figure 2 shows a plan-view optical image of the sample after all the microfabrication processes (as in Figure 1d). As shown in Figure 2, distinct (Au)-Si-Ge micro- and nanostructures were clearly observed under a plan-view optical microscope for two different micro-crucible geometries: vertical (left) and horizontal trapezoids (right). Effectively, the two had different sizes of openings (shown in red double arrows) that dictate the part of the liquid–vapour interface that was exposed to GeH_4_ during the CVD growth step (as previously described in Figure 1a,b). The vertical ones had smaller openings of ≅11.5 μm than the horizontal ones (37.4–47.3 μm). Both openings had a height of ≅0.1 μm, which made the liquid–vapour interfaces 1.15 μm^2^ and 3.74–4.73 μm^2^ for small and large openings, respectively. The residual micro- and nanostructures of the micro-crucibles with large openings appear to be less regular than the micro-crucibles with small openings. The micro-crucibles with larger openings look to have lighter dendritic-shaped remaining. This geometrical effect is so strong that it was observed in neighbouring micro-crucibles (shown in Figure 2) which are only ≅125.68 μm apart. In addition, this trend was observed across the whole sample with the dimension of 8 × 8 mm^2^.

**Figure 3:**
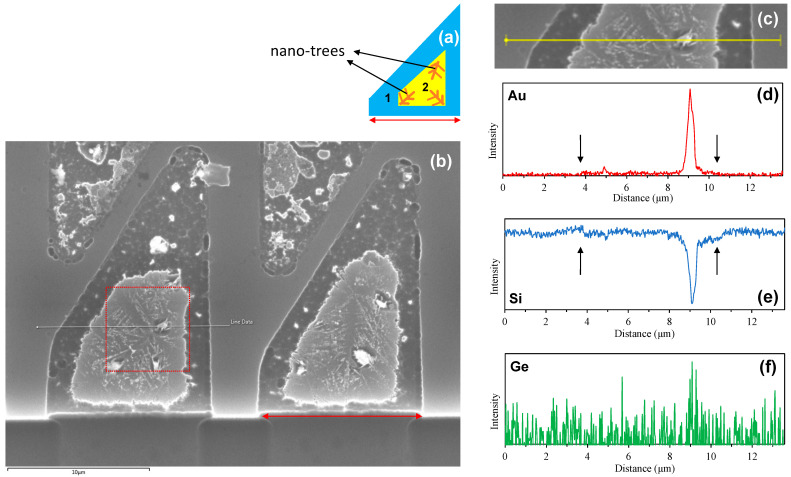
(**a**) Plan-view schematic of the sample with a small opening (shown by a red double arrow) with two regions: (1) dark outer and (2) bright inner regions that have nano-trees. (**b**) Plan-view SEM micrograph of micro-crucibles with a small opening showing the formation of nano-trees in region 2. The white line is an EDS line scan trajectory, cropped and realigned in (**c**). The dotted red box is a region of interest that will be analyzed later in an AFM analysis. The corresponding EDS line scan spectra of Au, Si, and Ge species are shown in (**d**), (**e**), and (**f**), respectively.

Further structural analyses were carried out by SEM, EDS, and AFM. Figure 3a shows a plan-view schematic of a micro-crucible with a small opening, indicated by a red double arrow. Figure 3b shows a plan-view SEM image of two neighbouring micro-crucibles with small openings. As seen, there are brighter (which are located in the middle, inner part of the trapezoid) and darker outer regions of the micro-crucible which may correspond to either the difference in the height or chemical composition (or both). These two regions are schematically shown in Figure 3a and labeled as region 2 and 1, respectively. Moreover, eutectic lamella “nano-trees” can be observed in region 2 of the sample. An EDS line scan was executed across the micro-crucible along the white line shown in Figure 3b with the solid circle (left) showing the starting point of the scan. Figure 3c shows the cropped and realigned image of the region of interest.

**Figure 4:**
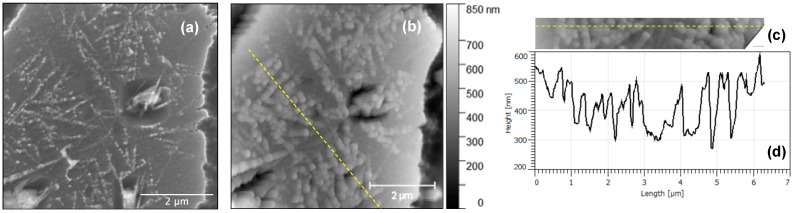
(**a**) Plan-view SEM micrograph of a region with nano-trees (a micro-crucible with a small opening) and (**b**) an AFM micrograph of the same region. The dashed yellow line shows a region of interest for a height profile analysis, cropped and realigned in (**c**), in which the resulting height profile is shown in (**d**).

The resulting EDS spectra indicate the presence of residual Au species in the middle (bright) part of the micro-crucible as shown in Figure 3d. This suggests that the bright white spots in the SEM image (Figure 3b) are residual Au regions that were not well etched or alloyed with the Ge (and Si). This is supported by the fact that the Si spectra, shown in Figure 3e, have a reciprocal relationship with the Au spectra (i.e., a trough on the particulate location) or a smaller decrease across the region 2 (pointed out by arrows in Figure 3e which has a small increase in Au shown in Figure 3d). This could happen because the presence of the Au attenuates the Si signal from the underlying Si substrate as is expected with atoms with a high atomic number [19] (this is further substantiated later by AFM analysis, in that the Au particles in Figure 3 and Figure 4 are not substantially thicker than the neighbouring nano-trees which are no more than 1 μm different, as shown later in Figure 4b).

Meanwhile, the Ge spectra, shown in Figure 3f, show a very noisy signal. This noisy spectrum suggests the presence of Ge species even in locations that were fully capped with SiO_2_/Si_3_N_4_ (not micro-crucibles) where Ge atoms could not reach (i.e., a distance of ≅1 μm and ≅13 μm). Using them as the background noise levels suggests a negligible amount of Ge presence in the micro-crucible. This observation suggests that the Ge species were also removed as the Au was etched. In other words, all Ge species were contained as a part of the Au-Si-Ge eutectic alloy, not nucleated as Ge crystallites on the Si substrate via the VLS process. This idea is further supported by the fact that the noisy EDS spectra of Ge have a maximum value at ≅9 μm which coincides with the location of the Au peak. In addition, we confirmed that the Transene Gold Etchant TFA solution used in the Au removal step has a high etching selectivity of 20 between Au and Ge (i.e., 28 Å/s and 1.4 Å/s for Au and Ge, respectively). This means that if Ge nucleated on the Si substrate by the VLS mechanism, the thin Ge layer would stay after dipping the sample into the Transene Gold Etchant TFA solution, provided the Ge layer has a thickness larger than 7.7 nm (i.e., 1.4 Å/s × 55 s).

Next, AFM analysis was carried out to further investigate the surface topography of the micro-crucible especially the distinct nano-trees observed in the SEM. For this reason, we carried out further analysis on the region shown in the dotted red box shown in Figure 3b. Figure 4a shows a higher resolution SEM image of this region, the dotted red box in Figure 3b. The AFM micrograph of the same region is shown in Figure 4b. Although there is an apparent width difference between images from SEM and AFM, the height contrast from AFM is still accurate. The wider appearance of the nanostructure features under AFM (than under SEM) is known as dilation artefacts due to a finite radius of a probe [20]. It will be further confirmed later in Figure 6b that the nano-pits (holes) appeared to be smaller under AFM than under SEM (an opposite effect). By comparing the SEM and the AFM images, we can see that the surface is very rough with the formed nano-trees being higher than the background surface. In addition, we can see that the origin of the contrast as seen in the SEM image between region 2 (where the nano-trees are located) and region 1 can be at least partially attributed to the height difference of up to 0.5 μm. This height difference is possibly due to the agglomeration of the Au(-Si) eutectic liquid droplet during CVD growth [16]. Region 2 was the location of this Au(-Si) droplet, which after the CVD was completed became solidified Au-Si-Ge alloy. The Si under the Au regions were well protected during the SF_6_-based DRIE step (Figure 1c), while the Si substrate in region 1 was exposed and thus etched by SF_6_ gas [18] as described previously in Figure 1c. This created a height difference between region 1 and 2, as schematically shown in Figure 3a. Together with the EDS results (i.e., a small increase in the Au signal across region 2 as shown in Figure 3d), we can deduce that both height and elemental differences are responsible for the SEM contrast we observe between regions 1 and 2 in Figure 3b). Moreover, in region 2, the formed nano-trees have a linear density of ≅2.25 μm^−1^. In addition, a further height profile analysis was conducted on a region of the micro-crucible indicated by a dashed yellow line in Figure 4b, cropped and realigned in Figure 4c. The height profile of the region, shown in Figure 4d, suggests that the nano-trees are formed on the substrate with a thickness as large as 270 nm.

**Figure 5:**
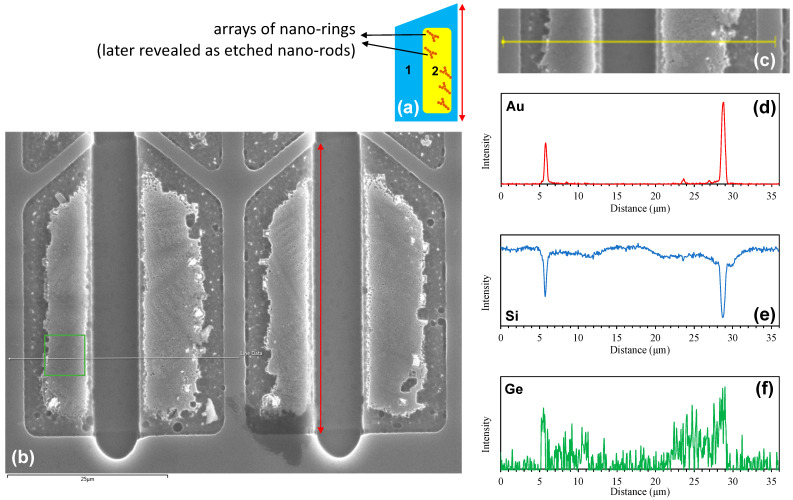
(**a**) Plan-view schematic of the sample with a large opening (shown by a red double arrow) with two regions: (1) dark outer and (2) bright inner regions that have arrays of nano-rings (later revealed as etched nano-rods). (**b**) Plan-view SEM micrograph of micro-crucibles with a large opening showing the formation of arrays of nano-rings in region 2. The white line is an EDS line scan trajectory, cropped and realigned in (**c**). The corresponding EDS line scan spectra of Au, Si, and Ge species are shown in (**d**), (**e**), and (**f**), respectively.

Similarly, we investigated the structural properties of the micro-crucibles with a large opening (horizontal trapezoids in Figure 2) using SEM, EDS, and AFM. Figure 5b shows the obtained SEM image showing four micro-crucibles with a schematic shown in Figure 5a. Note that the horizontal trapezoids were rotated counterclockwise by 90° (this was done to suppress the shadowing effects associated with the location of the EDS detector, i.e., located on the middle top of Figure 5b, when completing the EDS line scan later). As shown, for each micro-crucible, there is a brighter region in the inner part (as was the case of micro-crucibles with a small opening), called region 2 in Figure 5a which indicates either topographic or/and chemical composition differences. This region contains arrays of nano-rings that previously look dendritic in the optical micrograph in Figure 2 (we will come back to this discussion shortly). 

Then, an EDS line scan was executed across two neighbouring micro-crucibles (with a large opening) along the white line shown in Figure 5b with the solid circle showing the starting point of the scan. Figure 5c shows the cropped and realigned image of the region of interest. The EDS analyses reveal the presence of two strong Au peaks in the Au spectra (Figure 5d) in locations coinciding with Si troughs in the Si spectra (Figure 5e). The SEM image (Figure 5b cropped and realigned in Figure 5c) suggests that these Au peaks correspond to the existence of remaining Au particulates which appear as bright white spots in the SEM image. As before, the reciprocal relationship between Au and Si indicates that the Au particulates interfere with the EDS spectra of the underlying Si substrate due to its much larger atomic number than that of Si (hence, reducing the EDS signal that comes from the underlying Si substrate) [19]. Interestingly, the EDS line scan strongly suggests that region 2 of the micro-crucible contains Ge species in it, as shown in Figure 5f. This is different from the micro-crucibles with a small opening in which a negligible EDS signal of Ge was observed (either due to a thinner solidified Ge layer on the Si substrate that was etched away in a shorter time after dipping it into the etchant or because the Ge was only embedded in the (Au-rich) Au-Si-Ge eutectic solid which was removed by the etchant). In addition, as before, the EDS spectra of Ge have peak values that coincide with the location of the Au peaks which suggests a contribution of residual Ge species from the etched Au-Si-Ge eutectic.

**Figure 6:**
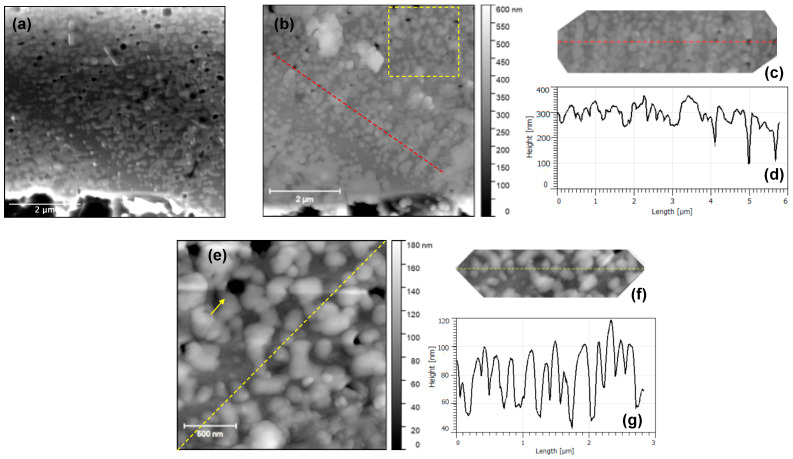
(**a**) Plan-view SEM micrograph of a region with arrays of nano-dots (a micro-crucible with a large opening, the solid green box in Figure 5b). (**b**) AFM micrograph of the same region in (**a**). The dashed red line shows a region of interest, cropped and realigned in (**c**) with the resulting height profile shown in (**d**). (**e**) A higher resolution AFM micrograph of the region in the dashed yellow box in (**b**). The yellow arrow highlights the formation of ring-like structures, i.e., when several nano-dots agglomerate with a pit presence in the middle. Meanwhile, the dashed yellow line shows a region of interest for a height profile analysis, cropped and realigned in (**f**), in which the resulting height profile is shown in (**g**).

Figure 6a shows a higher resolution SEM image of region 2 (where the Au was agglomerated and then formed a Au-Si-Ge eutectic alloy [16]) inside the solid green box in Figure 5b. As seen, a number of nano-dots (brighter contrast) are observed inside the bright inner region (i.e., region 2). In addition, we can observe the formation of darker circles (nano-pits) with various inner diameters of up to 275 nm. To confirm the topographic nature of these nanostructures, AFM analyses were conducted. Figure 6b shows the AFM micrograph of the same region as in Figure 6a. The AFM image confirms that the height difference is at least partially responsible for the contrast we observed in the SEM image with the darker circle regions in the center being recessed and effectively forming a pit in the center of the brighter (higher) nano-dots that are agglomerated and form a ring-like structure. Further height profile analyses, on the red, dashed line in Figure 6b (cropped and realigned in Figure 6c), suggest that the pits have a depth of up to 200 nm with respect to the surrounding nano-dots, as shown in Figure 6d. Figure 6e shows a higher-resolution AFM micrograph that corresponds to the region indicated by the dashed yellow box in Figure 6b. The yellow arrow in the figure points to one pit (i.e., a ring-like structure) that is surrounded by several nano-dots. We can see that not all nano-dots form ring-like structures with a pit in the middle. These nano-dots appear to be so dense in this region with an area density of 12.20 μm^−2^. A further height profile analysis revealed that such nano-dots have a height of 20–60 nm, as shown in Figure 6f,g.

**Figure 7:**
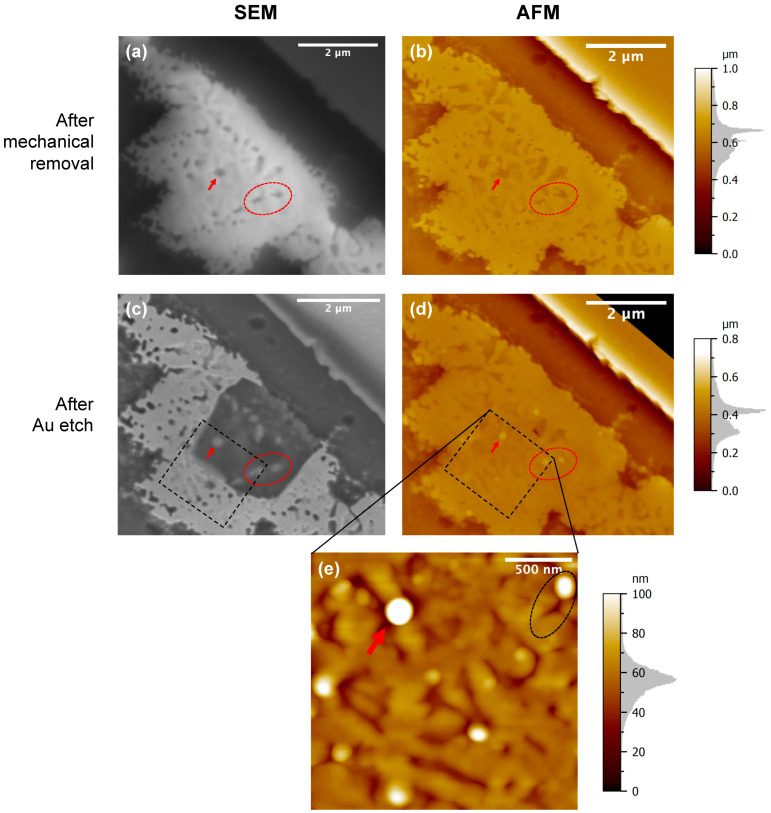
(**a**) Plan-view SEM micrograph of the solidified Au-Si-Ge eutectic from a large-opening micro-crucible after its capping layer was mechanically removed. (**b**) AFM micrograph of the same region. (**c**) Plan-view SEM micrograph of the same region after the sample was dipped into a Transene Gold Etchant TFA solution for 55 s. (**d**) AFM micrograph of the same region. (**e**) A higher resolution AFM micrograph of the region inside the dashed black box in (**c**,**d**). The red arrow and the dashed red oval in all figures correspond to the same region of nano-rods.

The formation of regions 1 and 2 which have a substantial topographic contrast in both micro-crucible types (Figure 3b and Figure 5b) suggests that the SF_6_-based DRIE step (Figure 1c) carried out to remove the capping layer made of SiO_2_/Si_3_N_4_ might also etch the semiconductor-rich phase (i.e., SiGe) of the solidified Au-Si-Ge eutectic. In fact, SF_6_ has been used to etch both Si and Ge [18]. Hence, to understand the origins of the previously observed nanostructures, we used Scotch tape (instead of the DRIE step) to adhere and mechanically remove the capping layer. This way, the semiconductor-rich phase could be preserved in the eutectic structure. Figure 7a shows a plan-view SEM micrograph of the solidified Au-Si-Ge eutectic from a large-opening micro-crucible after its capping layer was mechanically peeled off. We can observe the formation of darker contrast arrays of rods and lamella (i.e., laterally wider rods) in a Au-rich matrix. This darker contrast can be partially attributed to the compositional nature of the phase which is semiconductor rich (i.e., both Ge and Si have much smaller atomic numbers than that of Au). In addition to the compositional difference, the topographical difference is also responsible for the contrast we observed in the SEM. Figure 7b shows the AFM micrograph of the same region (with the red arrow and dashed oval highlighting the same nanostructures), which suggests that the arrays of rods and lamella are lower than the Au-rich matrix.

Interestingly, from the plan-view, these arrays of dots and lamella have very similar shapes to the arrays of pits shown previously in Figure 6a. To understand them better, we also dipped the sample (with the capping layers mechanically peeled off) into a Transene Gold Etchant TFA solution for 55 s (same as before). Figure 7c,d shows the plan-view SEM and AFM micrographs of the sample after the Au etching, respectively. We can observe that a part of the Au-rich matrix in the middle was removed with a squarish shape from the Au-Si-Ge eutectic, revealing the underlying Si substrate. More importantly, the arrays of rods and lamella are preserved on the Si substrate. From the AFM micrograph in Figure 7d, we observe these nanostructures (highlighted by a red arrow and dashed oval) are now higher than the substrate, which further confirms the formation of the semiconductor-rich phase since Transene Gold Etchant TFA solution is fairly selective to Au compared to Si and Ge. Moreover, a high-resolution AFM, shown in Figure 7e, suggests that the lamella nanostructure previously shown in the SEM in Figure 7c is actually composed of a rod followed by a short canal (a rod + a canal = a lamella under SEM) as shown in the dashed black oval in Figure 7e. This canal exposes the underlying Si substrate and hence, appears darker under the SEM. This finding suggests that the semiconductor-rich phase of the eutectic mostly has a rod-type morphology which implies that the volume fraction of two solid phases in our eutectic system is <0.32 [21]. From Figure 7e, we also found that the rod can be as thick as 72.3 nm (i.e., the one pointed out by the red arrow).

**Figure 8:**
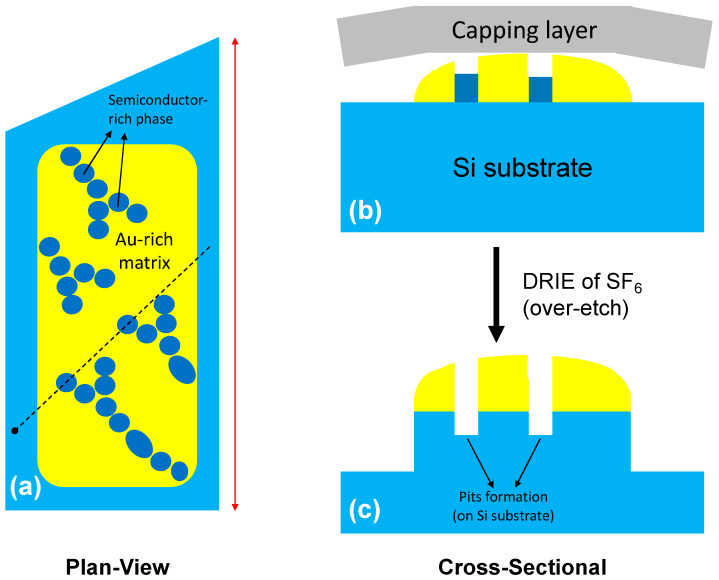
Proposed schematic of the pit formation process: (**a**) plan-view schematic of the sample with a large opening (indicated by a red double arrow) showing the formation of arrays of rods and lamella which are semiconductor-rich phases in the vicinity of the Au-rich matrix. The dashed line indicates the cross-sectional line shown in (**b**,**c**) for the schematics before and after the SF_6_-based DRIE step, respectively. The solid circle of the line indicates the left-most region in (**b**,**c**).

**Figure 9:**
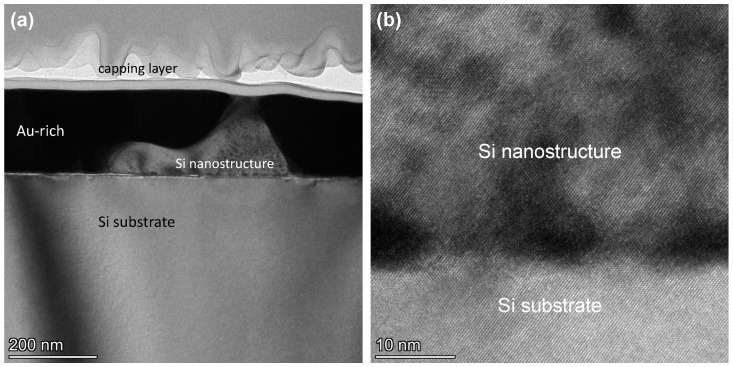
(**a**) A cross-sectional TEM image showing the formation of a Si nanostructure. (**b**) A higher resolution TEM image of the formed nanostructure showing its epitaxial relationship with the underlying Si substrate.

The fact that the shapes of the arrays of the rods (and lamella) are similar to the formed pits in the sample that underwent the SF_6_-based DRIE step (Figure 6a) clarifies the mechanism of the pit formation, as schematically shown in Figure 8. For micro-crucibles with a large opening, the semiconductor-rich phase of the Au-Si-Ge eutectic has a rod (and lamella) shape in the vicinity of the Au-rich matrix (Figure 8a). This semiconductor-rich phase is slightly lower than the Au-rich phase with a regular alignment between its top-most and bottom-most parts (Figure 8b). Due to the compositional nature of this semiconductor phase (mostly Si and/or Ge), it was etched during the SF_6_-based DRIE. As a result, parts of the underlying Si substrate where the rods (and lamella) were located were also etched by SF_6_. However, other parts of the Si substrate with the Au-matrix on top (which acted as the etching mask) were preserved after this long DRIE step. Therefore, pits were formed only on the locations of the rods (and lamella). The absence of any pits from the Au-Si-Ge eutectic grown from the small-opening micro-crucibles (Figure 4a) suggests that the top- and bottom-most parts of the semiconductor-rich phase of the eutectic were not well aligned. This was possibly due to a more irregular shape of the phase, which was related to a different Ge content in the solidified Au-Si-Ge eutectic (less Ge in small-opening micro-crucibles). Figure 9a shows a cross-sectional TEM image (prepared using an FIB) from a micro-crucible without any exposures of Ge precursors (effectively long annealing). The capping layer was removed by neither DRIE nor mechanical peeling. From Figure 9a, we can observe the formation of Si “hillock” nanostructures in the vicinity of the Au-rich phase. As there was not any external supply of any Si precursors during the annealing, the Si atoms that make up the nanostructure came from the Si substrate that acted as a Si reservoir during the annealing process. Importantly, the formed Si nanostructure has an epitaxial relationship with the underlying Si substrate, as shown in a higher-resolution TEM in Figure 9b. 

**Figure 10:**
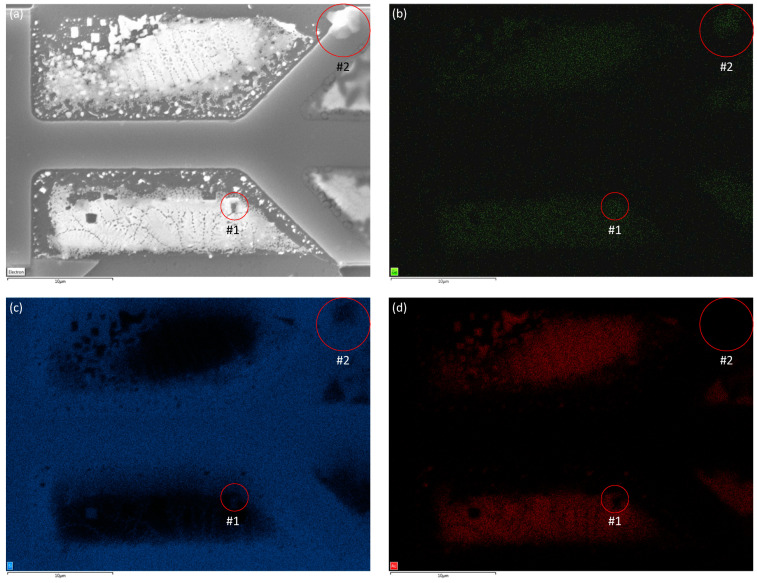
(**a**) Plan-view SEM micrograph of two neighboring large-opening micro-crucibles and the corresponding EDS maps of (**b**) Ge, (**c**) Si, and (**d**) Au.

The presence of Ge, as indicated by the EDS data in Figure 5f, on the micro-crucibles with a large opening suggests the possible nucleation of Ge crystals in this type of micro-crucible using the CVD growth conditions (4 sccm of GeH_4_, 40 mTorr, and 375 °C for 1 h). As opposed to the micro-crucibles with a small opening, the micro-crucibles with a large opening have a large enough liquid–vapour interface in which the GeH_4_ precursor gas could crack on the surface of the liquid droplets of Au-Si eutectic. In fact, this Ge nucleation exists and a lateral Ge film with an area of ≅0.4 μm^2^ (as seen from the top view) was observed as shown inside the red circle #1 in Figure 10a which coincides with Ge-rich region #1 in the EDS map of Ge shown in Figure 10b. Note that Ge-rich region #2 in Figure 10b corresponds to a Ge film grown (via uncatalyzed vapour–solid) on top of the capping layer. As shown in Figure 10c, the laterally grown Ge film (red circle #1) has some Si content in it. This has also been reported before [16] and happened due to the strong solubility of Si and Ge. Moreover, the Ge-rich region #1 has a very weak Au signal, as shown in Figure 10d, which further confirms that the strong Ge signal came from nucleated Ge (not from the Au-Si-Ge eutectic). Figure 10c,d also reveals the presence of the rod (and lamella) nanostructures which are Si-rich, similar to what we have found before.

In micro-crucibles with a small opening, the liquid–vapour interface is likely too small to induce a supersaturation state of the Au-Si-Ge eutectic alloy under the growth conditions and hence, no nucleation of Ge crystallites [14]. This observation of a strong suppression on the nucleation of the VLS growth at the micro-scale by means of geometric confinement (i.e., reducing the dimension of the liquid–vapour interface, where the precursor gases could reach and dissolve in the Au catalyst) is interesting as previously we only considered the nano-scale VLS in which the “geometric suppression” is predominantly due to the Gibbs–Thomson effect [8,9]. This observation is also in good agreement with the previous observations through kinetics data collected by plan-view SEM measurements in which micro-crucibles with double openings (effectively with a larger net liquid–vapour interface) resulted in a larger Ge micro-films area after the CVD growth step (as measured in the plan-view) [11]. The CVD growth conditions used in that experiment were similar to the present experiment, but with a larger GeH_4_ flow rate (5 sccm [11] compared to the present GeH_4_ flow of 4 sccm). Hence, the lateral Ge micro-films obtained in that experiment could be observed more easily from plan-view SEM as they are thicker than the case in the present study. With the present CVD growth conditions, it would require more “incubation” time for micro-crucibles with a small opening to nucleate Ge crystallites [22]. Therefore, these observations suggest the presence of geometrical effects on the incubation time for VLS nucleation.

To understand and quantify this geometric suppression of Ge crystallization, we consider the incubation time required to reach supersaturation in the liquid Au-Si-Ge alloy [22]. Using a modified approach of Ref. [14], the number of Ge atoms in the alloy changes with time according to:

(1) $$\frac{dN_{Ge}}{dt}=\left(I-I_{des}^{Ge}x_{Ge}\right)HL$$

Here, I is the incoming Ge vapour precursor flux into the alloy and $I_{des}^{Ge}x_{Ge}$ is the desorption flux of Ge atoms. In contrast to Ref. [14], the desorption flux is taken proportional to Ge content in the alloy, $x=N_{Ge}/\left(N_{Au}+N_{Si}+N_{Ge}\right)$, with $N_{k}$ as the numbers of atoms *k* = Au, Si, and Ge, and $I_{des}^{Ge}$ as a temperature-dependent pre-factor. This representation is valid because the GeH_4_ vapour precursor contains one Ge atom [23]. Both fluxes enter or leave the alloy through the liquid–vapour interface area HL, where H≅ 100 nm is the height and L is the length of the opening (≅11.5 μm for the small vertical openings or 37.4–47.3 μm for the large horizontal openings). Assuming that $N_{Ge}\ll N_{Au}+N_{Si}$, we can treat $N_{tot}=N_{Au}+N_{Si}+N_{Ge}$ as a time-independent value. In this case, Equation (1) is reduced to:

(2) $$\frac{dx_{Ge}}{dt}=\frac{x_{*}-x_{Ge}}{\tau }$$

with $x_{*}=I/I_{des}^{Ge}$ and $\tau =V/\left(I_{des}^{Ge}\Omega HL\right)$. Here, *V* is the total volume of the alloy in the micro-crucible and Ω is the elementary volume in liquid. 

Solving Equation (2) with the initial condition $x_{Ge}\left(t=0\right)=0$, we obtain:

(3) $$\;x_{Ge}=x_{*}\left(1-e^{-t/\tau }\right)$$

Therefore, $\;x_{Ge}$ tends to $x_{*}$ with the characteristic time constant τ. The VLS nucleation of Ge crystallites from the alloy is possible only when its content in the alloy is larger than the equilibrium content $x_{eq}$. From Equation (3), reaching $x_{eq}$ requires the incubation time:

(4) $$\Delta t_{inc}=\frac{V}{I_{des}^{Ge}\Omega HL}\left[-ln\left(1-\frac{I_{des}^{Ge}x_{eq}}{I}\right)\right]$$

At a fixed volume and height of the micro-crucible (*V* = const and *H* = const), growth temperature ($I_{des}^{Ge}=$ const and $x_{eq}=$ const), and GeH_4_ flow rate (*I* = const), the incubation time scales is $L^{-1}$, that is, time is longer for smaller openings. Our large openings have 3.25-4.11 times greater L compared to the smaller ones. If the volume of the crucibles varies, the parameter which determines the incubation time in Equation (4) is the geometric ratio a=V/(HL). Measurements of different micro-crucibles give the mean a of 15.95 μm for small openings and 12.55 μm for large openings (see Supplementary Material). Therefore, the average incubation time is ≅27% longer in the micro-crucibles with small openings. This explains why Ge crystallites nucleated in the micro-crucibles with large openings and did not emerge in the micro-crucibles with small openings after the same deposition time of Ge (1 h). 

It is interesting to note that, at $I_{des}^{Ge}x_{eq}\ll I$, Equation (4) is reduced to:

(5) $$\Delta t_{inc}=\frac{c_{eq}}{I}\frac{V}{{HL}^{\prime }}$$

where $c_{eq}=x_{eq}/\Omega$ is the dimensional equilibrium concentration of Ge in μm^−3^. This result follows from Ref. [14] assuming negligible desorption. Otherwise, Equation (4) generalizes the earlier result [14] to the case of composition-dependent desorption rate. From Equations (4) and (5), it also follows that the incubation time is shorter for larger Ge fluxes I, which is expected. 

## 4. Conclusions

In conclusion, novel semiconductor nanostructures were obtained via a four-step procedure which includes patterning of a capping layer on a Si(100) substrate, deposition of the Au layer, annealing and CVD growth of Ge via the VLS process, DRIE (can also be a mechanical peeling) to remove the capping layer, and etching to remove Au. The morphology and composition of the resulting nanostructures strongly depends on the size of the opening in the capping layer which determines the area of the liquid–vapour interface in the Ge deposition step. It has been shown that the presence or absence of VLS nucleation of Ge crystallites in such a process can be tuned by controlling the size of the opening. The incubation time for the VLS nucleation of Ge from the liquid Au-Si-Ge alloy is shorter for larger openings, which is why no Ge crystallites nucleate in micro-crucibles with a smaller opening size. This geometric suppression of VLS nucleation can be used in different material systems provided that the size of the growth template exposed to vapour can be defined by photolithographic patterning. By changing the opening size, one can fabricate VLS nano- or micro-crystals with different sizes, shapes, and compositions, which gives an additional tuning knob for obtaining structures with the desired properties.

## Data Availability

Not applicable.

## Supplementary Materials

The following supporting information can be downloaded at: https://www.mdpi.com/article/10.3390/nano13050894/s1, Table S1. Measurements of geometric ratios for micro-crucibles with small openings. Table S2. Measurements of geometrics ratio for micro-crucibles with large openings.
