# Supplementary material for: Tuning the Liquid–Vapour Interface of VLS Epitaxy for Creating Novel Semiconductor Nanostructures
Source: Nanomaterials (Basel). 2023 Feb 27;13(5):894. doi: 10.3390/nano13050894 (PMC10005286; doi:10.3390/nano13050894)
Supplement: Supplementary file 1 [file nanomaterials-13-00894-s001.zip › nanomaterials-2209048-supplementary.pdf]

nanomaterials

MDPI

Supplementary Materials

Tuning the Liquid–Vapour Interface of VLS Epitaxy for Creating Novel Semiconductor Nanostructures

Galih R. Suwito [1], Vladimir G. Dubrovskii [2], Zixiao Zhang [3], Weizhen Wang [1], Sofiane Haffouz [4], Dan Dalacu [4], Philip J. Poole [4], Peter Grutter [3] and Nathaniel J. Quitoriano [1,*]

1 Department of Mining and Materials Engineering, McGill University, Montreal, QC H3A 0C5, Canada
2 Faculty of Physics, St. Petersburg State University, St. Petersburg 199034, Russia
3 Department of Physics, McGill University, Montreal, QC H3A 2T8, Canada
4 National Research Council Canada, Ottawa, ON K1A0R6, Canada
* Correspondence: nate.quitoriano@mcgill.ca

Geometric ratio α is defined as:

$$\alpha \equiv \frac{V}{H.L}, \qquad \text{(S1)}$$

where V is the total volume of the alloy in the micro-crucible, H is the thickness of the alloy, and L is the length of the opening. With the known plan-view area of the alloy in the micro-crucible A_{cru}, the geometric ratio α can be estimated assuming that the thickness H is uniform across the micro-crucible:

$$\alpha \equiv \frac{A_{cru}.H}{H.L} = \frac{A_{cru}}{L}. \qquad \text{(S2)}$$

Hence, by measuring the length of the opening L and the plan-view area of the alloy A_{cru}, we can estimate the values of the geometric ratio. Tables S1 and S2 show the measurements of the geometric ratios of micro-crucibles with small and large openings, respectively.

Table S1. Measurements of geometric ratios for micro-crucibles with small openings.

No	L (μm)	A_{cru} (μm^2)	α (μm)
1	11.79	187.25	15.89
2	11.68	177.91	15.23
3	11.85	166.76	14.07
4	11.65	156.11	13.40
5	11.65	145.15	12.46
6	11.77	132.76	11.28
7	11.46	243.03	21.21
8	11.65	227.30	19.51
9	11.72	217.28	18.54
10	11.79	210.60	17.87
		**Mean α**	15.95

Table S2. Measurements of geometrics ratio for micro-crucibles with large openings.

No	L (µm)	A_{cru} (µm²)	α (µm)
1	38.86	481.22	12.38
2	45.32	458.78	10.12
3	37.51	583.23	15.55
4	47.21	576.10	12.20
5	39.66	486.86	12.27
6	45.52	461.33	10.13
7	37.44	579.98	15.49
8	47.34	578.01	12.21
		**Mean α**	12.55
